# Surgical removal of a tea spoon from the ascending colon, ten years after ingestion: a case report

**DOI:** 10.4076/1757-1626-2-7532

**Published:** 2009-09-09

**Authors:** Samer Deeba, Sanjay Purkayastha, San Jeyarajah, Ara Darzi

**Affiliations:** Department of BioSurgery & Surgical Technology, Imperial College Healthcare, St. Mary’s Hospital CampusQEQM Building, 10^th^ Floor, South Wharf Road, London W2 1NYUK

## Abstract

**Introduction:**

The presentation of ingested foreign bodies in the gastrointestinal system is common in the emergency setting. The majority responds to conservative management and passes spontaneously; however, giant foreign bodies pose a management difficulty. We report a peculiar case of a giant foreign body (spoon) that presented very late after ingestion and the management of this presentation.

**Case presentation:**

A 30-year-old British white male barrister presented with abdominal pain 10 years after he swallowed a spoon that never passed spontaneously. His workup revealed the spoon lodged in his ascending colon. Laparoscopic retrieval was not feasible so a laparotomy was done for retrieval. He did well and went home with no complications.

**Conclusion:**

Symptomatic giant ingested foreign bodies represent a management challenge sometimes and usually necessitate surgical intervention when all conservative means fail. We review the literature on management of giant ingested foreign bodies.

## Case presentation

**Figure 1.**
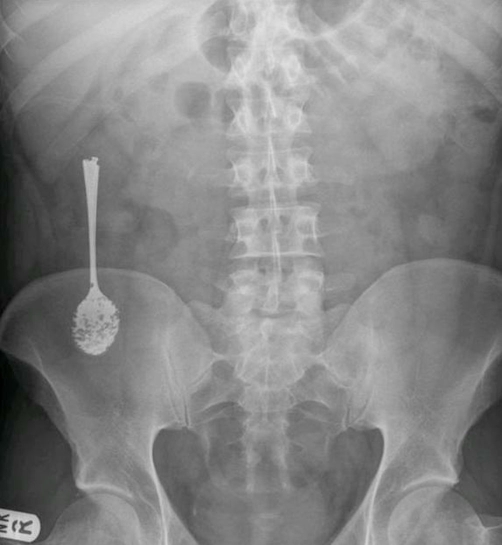
Plain abdominal X-ray.

**Figure 2.**
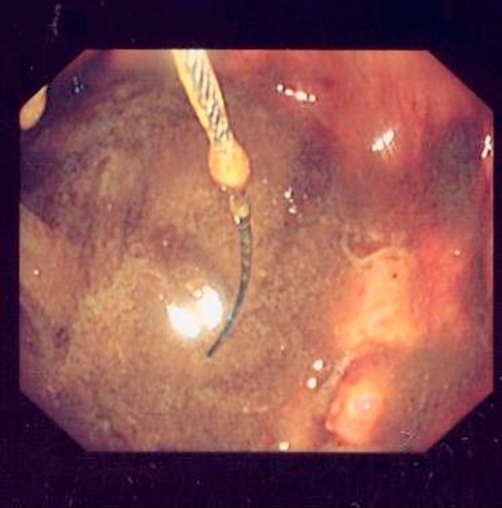
Endoscopic image of the spoon in the ascending colon.

In this article we present an unusual case of ingested giant foreign body that necessitated surgical intervention for its retrieval. A previously known fit and healthy British white 33-year-old male presented 10 years after he swallowed two tea-spoons while he was under the influence of alcohol. He passed one spontaneously per rectum a year after ingestion, but the other never passed. A subsequent CT scan showed the remaining spoon still in the ascending colon and was reported as being intraluminal. He was lost to follow up until he started experiencing some vague abdominal pain in the right iliac fossa intermittently which he addressed with regular over-the-counter analgesia and that managed his symptoms adequately at first. He never experienced severe pain, nausea or vomiting, neither the passage of any blood per stool. His laboratory profile seems within normal with a hemoglobin of 13.8 and white blood cells of 7.2 and C-reactive protein of less than 5. His dietary intake and bowel habits were unchanged. A plain abdominal X-ray confirmed the presence of the spoon in the right iliac fossa (Figure 1). Subsequently, he was referred for an outpatient colonoscopy for further evaluation and attempt at retrieval. The procedure was not successful at removing the spoon, due to the adherence of the spoon to the colon wall, but confirmed its position in the ascending colon. Some minor inflammatory tissue and ulceration was seen in the colon wall at the two ends of the spoon hence hindering adequate snaring and retrieval also (Figure 2). The patient was subsequently seen in clinic and as he felt so symptomatic, where the pain has been increasing over the last few months prior to presentation and became refractory to over the shelf analgesia, was scheduled for a laparoscopic limited right hemicolectomy.

**Figure 3.**
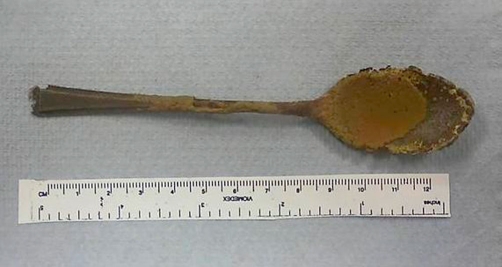
The spoon after retrieval.

**Figure 4.**
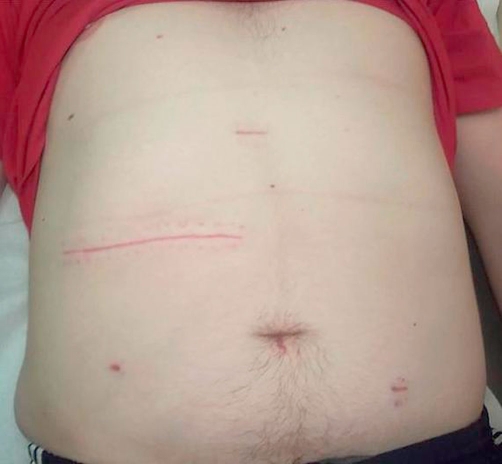
Follow up in 2 months.

Intraoperatively, open Hasson port pneumoperitoneum was achieved but initial laparoscopy revealed dense adhesions of the ascending colon to the anterior abdominal wall with the ascending colon and hepatic flexure very adherent to the retroperitoneum hindering adequate exposure and tactile feedback of the spoon’s position. There was also thick scar tissue and edema rendering the lateral side of the right colon adherent to the abdominal wall; and this is probably due to micro-perforations at the anterolateral border of the proximal ascending colon. In view of these findings the procedure was converted to a laparotomy through a right transverse incision where open lysis of adhesions was safely and meticulously carried out revealing the position of the spoon confirmed by manual palpation. A colotomy was performed, the spoon delivered and extracted. The colotomy was closed using a two layer technique. The spoon measured 13.5 centimeters long and 2.5 centimeters wide (Figure 3). The patient did well postoperatively with no subsequent complications and was discharged home three days later. The patient returned for follow up two months after surgery and was symptom free and doing very well (Figure 4).

## Conclusion

Foreign body (FB) ingestion is a common presentation to the surgical team, usually as children accounting for about 80%; the remainder usually including prisoners, psychiatric patients, alcoholics, and senile patients [1,2]. Most ingested FBs pass through the GI tract without clinical consequences; however in about 10-20% endoscopic removal is warranted and 1% require surgical intervention [3]. In the oesophagus there are three anatomical narrowing where FBs may become lodged: the upper and lower oesophageal sphincters and where the aortic arch crosses over. There is a more than 90% chance that a FB is passed spontaneously once it reaches the stomach. However, objects larger than two centimeters in diameter also may lodge at the pylorus; whereas objects longer than six centimeters may become entrapped at the pylorus or at the C-curve of the duodenum between the first, second and third parts of the duodenum and rarely pass beyond that [4,5]. Other than this, the only remaining obstacle against the passage of the FB is the ileocecal valve. Rarely, a foreign body becomes entrapped in a Meckel’s diverticulum or at the sigmoid S-curve which is more flexible than the duodenal C-curve since it is not fixed in the retroperitoneum and hence can give way to the passage of the FB [6]. In the case presented above there is a rare presentation where a spoon longer than 6 centimeters and wider than 2 centimeters has passed all the way down and lodged the ascending colon due to erosion and adhesion into the colon wall (or probably could not negotiate its way through the valve of Gerlach (ileoceacal valve).

Complications of FB ingestion range from hemorrhage, bowel obstruction, perforation and erosion into adjacent viscera. Hemorrhage occurs when the FB injures the mucosa or lodges in a region close to visceral artery like in the pylorus eroding into the gastroduodenal artery. Bowel obstruction can also occur when the FB is bigger than any of the narrow anatomical areas of the intestinal tract explained above; hence lodging there and causing obstruction. This can progress to hemorrhage when it erodes into an artery or injures the mucosa. It can perforate freely in the peritoneum or extra-peritoneally when it reaches the distal rectum presenting with abscess and rectal bleeding [2].

Intra-abdominal perforations can be acute or chronic. They may present acutely with peritonitis when they perforate freely. A chronic perforation may present as an inflammatory mass or abscess. Certain perforations may be completely asymptomatic when the perforation has been sealed of by the body defense mechanism and inflammatory reaction, such as this presentation. It is not uncommon that the presentation of an ingested foreign body may be more indolent. The perforation can occur slowly when the FB gets stuck at anatomical angulations or narrowing such as the ileocecal valve and the sigmoid giving ample time for the omentum and the surrounding viscera to seal off the slowly occurring perforation. It may eventually result in the FB eroding into these structures with a large amount of inflammation accompanying this process. Furthermore, parts of the duodenum and the colon are retroperitoneal and a perforation there may not present with the classical signs and symptoms of perforation but have a more subtle presentation [6]. In this case patients may present with pain probably due to sub-clinical perforation and then subsequent sealing off by the body defense mechanisms. However perforations of the jejunum and ileum tend to present acutely since they are intraabdominal and present as an abdominal crisis.

Sharp foreign bodies are the most challenging to manage; the most common are toothpicks, nails, bones, blades, teeth, dental prosthesis, pins, and needles. Amongst these the most common to require surgical intervention for extraction are toothpicks and bones [7]. Usually endoscopic removal of these is warranted and successful. Only about 1% perforate the gut and are all sharp and pointed FBs and should be removed before they pass the stomach since they are associated with a perforation at the ileocecal valve in about 40% of the cases [6]. The straight pin is an exception since the flow of the intestinal contents and the relaxation of the bowel wall tend to make the pin head lead and the sharp end trail behind; hence making it a safe passage. Once the pin is in the colon it gets engulfed by feces and passes safely [8]. As a general rule for management of ingested sharp objects, the patient is monitored and followed up for three days and if the object did not pass then surgical intervention is indicated [3,5].

We feel that laparoscopic procedures should be attempted in all such cases owing to its well documented advantages such as reduced postoperative pain, shorter hospital stay, decreased ileus, and faster return to regular activity. Laparoscopy can be used to retrieve small sharp objects like pins and blades or even larger longer objects like forks [9,10]. In this case laparoscopy was attempted however the extent of the adhesions and inflammation was overwhelming and a decision to convert was deemed safer for the patient.

In conclusion, ingested FBs in most scenarios pass spontaneously. The size and nature of the FB may be a limiting factor. Of those that do not pass spontaneously, about 20% are endoscopically removed from the stomach and duodenum. Only 1% cause acute presentations like perforations and obstructions and warrant surgical intervention. It is advisable that all patients with a history of ingested and non passed FBs be closely followed up, properly evaluated and surgery advised to prevent complications in the event that these FBs are not passed spontaneously within a year of ingestion.
